# A biomarker framework for liver aging: the Aging Biomarker Consortium consensus statement

**DOI:** 10.1093/lifemedi/lnae004

**Published:** 2024-01-30

**Authors:** Aging Biomarker Consortium, Mengmeng Jiang, Zhuozhao Zheng, Xuan Wang, Yanhao Chen, Jing Qu, Qiurong Ding, Weiqi Zhang, You-Shuo Liu, Jichun Yang, Weiqing Tang, Yunlong Hou, Jinhan He, Lin Wang, Pengyu Huang, Lin-Chen Li, Zhiying He, Qiang Gao, Qian Lu, Lai Wei, Yan-Jiang Wang, Zhenyu Ju, Jian-Gao Fan, Xiong Zhong Ruan, Youfei Guan, Guang-Hui Liu, Gang Pei, Jian Li, Yunfang Wang

**Affiliations:** State Key Laboratory of Membrane Biology, Institute of Zoology, Chinese Academy of Sciences, Beijing 100101, China; Institute for Stem Cell and Regeneration, Chinese Academy of Sciences, Beijing 100101, China; Department of Radiology, Beijing Tsinghua Changgung Hospital, School of Clinical Medicine, Tsinghua University, Beijing 102218, China; Hepatopancreatobiliary Center, Beijing Tsinghua Changgung Hospital, School of Clinical Medicine, Tsinghua University, Beijing 102218, China; CAS Key Laboratory of Nutrition, Metabolism and Food Safety, Shanghai Institute of Nutrition and Health, University of Chinese Academy of Sciences, Chinese Academy of Sciences, Shanghai 200031, China; State Key Laboratory of Stem Cell and Reproductive Biology, Institute of Zoology, Chinese Academy of Sciences, Beijing 100101, China; CAS Key Laboratory of Nutrition, Metabolism and Food Safety, Shanghai Institute of Nutrition and Health, University of Chinese Academy of Sciences, Chinese Academy of Sciences, Shanghai 200031, China; CAS Key Laboratory of Genomic and Precision Medicine, Beijing Institute of Genomics, Chinese Academy of Sciences and China National Center for Bioinformation, Beijing 100101, China; Department of Geriatrics, the Second Xiangya Hospital, and the Institute of Aging and Geriatrics, Central South University, Changsha 410011, China; Department of Physiology and Pathophysiology, School of Basic Medical Sciences, State Key Laboratory of Vascular Homeostasis and Remodeling, Center for Non-coding RNA Medicine, Peking University Health Science Center, Beijing 100191, China; The Key Laboratory of Geriatrics, Beijing Institute of Geriatrics, Institute of Geriatric Medicine, Chinese Academy of Medical Sciences, Beijing Hospital/National Center of Gerontology of National Health Commission, Beijing 100730, China; Yiling Pharmaceutical Academician Workstation, Shijiazhuang 050035, China; Department of Pharmacy, West China Hospital of Sichuan University, Chengdu 610041, China; Department of Hepatobiliary Surgery, Xijing Hospital, Fourth Military Medical University, Xi’an 710032, China; State Key Laboratory of Advanced Medical Materials and Devices, Engineering Research Center of Pulmonary and Critical Care Medicine Technology and Device (Ministry of Education), Institute of Biomedical Engineering, Chinese Academy of Medical Science & Peking Union Medical College, Tianjin 300192, China; Clinical Translational Science Center, Beijing Tsinghua Changgung Hospital, Tsinghua University, Beijing 102218, China; Institute for Regenerative Medicine, Shanghai East Hospital, School of Life Sciences and Technology, Tongji University, Shanghai Engineering Research Center of Stem Cells Translational Medicine, Shanghai 200092, China; Department of Liver Surgery and Transplantation, Liver Cancer Institute, Zhongshan Hospital, Fudan University, Shanghai 200032, China; Hepatopancreatobiliary Center, Beijing Tsinghua Changgung Hospital, School of Clinical Medicine, Tsinghua University, Beijing 102218, China; Key Laboratory of Digital Intelligence Hepatology (Ministry of Education), School of Clinical Medicine, Tsinghua University, Beijing 102218, China; Hepatopancreatobiliary Center, Beijing Tsinghua Changgung Hospital, School of Clinical Medicine, Tsinghua University, Beijing 102218, China; Department of Neurology, Daping Hospital, Third Military Medical University, Chongqing 400042, China; Key Laboratory of Regenerative Medicine of Ministry of Education, Institute of Aging and Regenerative Medicine, Jinan University, Guangzhou 510632, China; Department of Gastroenterology, Xinhua Hospital Affiliated to Shanghai Jiao Tong University School of Medicine, Shanghai 200092, China; Centre for Lipid Research & Key Laboratory of Molecular Biology for Infectious Diseases (Ministry of Education), Institute for Viral Hepatitis, Department of Infectious Diseases, the Second Affiliated Hospital, Chongqing Medical University, Chongqing 400016, China; Advanced Institute for Medical Sciences, Dalian Medical University, Dalian 116044, China; State Key Laboratory of Membrane Biology, Institute of Zoology, Chinese Academy of Sciences, Beijing 100101, China; Institute for Stem Cell and Regeneration, Chinese Academy of Sciences, Beijing 100101, China; University of Chinese Academy of Sciences, Beijing 100049, China; Collaborative Innovation Center for Brain Science, School of Life Science and Technology, Tongji University, Shanghai 200092, China; The Key Laboratory of Geriatrics, Beijing Institute of Geriatrics, Institute of Geriatric Medicine, Chinese Academy of Medical Sciences, Beijing Hospital/National Center of Gerontology of National Health Commission, Beijing 100730, China; Hepatopancreatobiliary Center, Beijing Tsinghua Changgung Hospital, School of Clinical Medicine, Tsinghua University, Beijing 102218, China; Clinical Translational Science Center, Beijing Tsinghua Changgung Hospital, Tsinghua University, Beijing 102218, China; Key Laboratory of Digital Intelligence Hepatology (Ministry of Education), School of Clinical Medicine, Tsinghua University, Beijing 102218, China; Research Unit of Precision Hepatobiliary Surgery Paradigm, Chinese Academy of Medical Sciences, Beijing 102218, China

## Abstract

In human aging, liver aging per se not only increases susceptibility to liver diseases but also increases vulnerability of other organs given its central role in regulating metabolism. Total liver function tends to be well maintained in the healthy elderly, so liver aging is generally difficult to identify early. In response to this critical challenge, the Aging Biomarker Consortium of China has formulated an expert consensus on biomarkers of liver aging by synthesizing the latest scientific literature, comprising insights from both scientists and clinicians. This consensus provides a comprehensive assessment of biomarkers associated with liver aging and presents a systematic framework to characterize these into three dimensions: functional, imaging, and humoral. For the functional domain, we highlight biomarkers associated with cholesterol metabolism and liver-related coagulation function. For the imaging domain, we note that hepatic steatosis and liver blood flow can serve as measurable biomarkers for liver aging. Finally, in the humoral domain, we pinpoint hepatokines and enzymatic alterations worthy of attention. The aim of this expert consensus is to establish a foundation for assessing the extent of liver aging and identify early signs of liver aging-related diseases, thereby improving liver health and the healthy life expectancy of the elderly population.

## Introduction

Aging refers to the pathophysiological process of gradual and irreversible degeneration of cells, tissues, and organs in the body, all of which leads to impaired function and high risk of death [1, 2]. During aging, the liver appears to be the only organ in the human body capable of resisting aging. In a study using retrospective radiocarbon (^14^C) birth-dating, hepatocytes self-renewed continuously, turning over throughout life regardless of donor age and thereby effectively allowing the liver to remain a young organ with an estimated average age of 3 years [3]. This unique organ biology suggests that an accurate assessment of true liver age could yield insights of both scientific and clinical significance, accompanied by substantial challenges. To date, a large number of biological and clinical studies have investigated liver function in the context of diseases and aging, but the field lacks a systematic analysis of liver aging biomarkers. Therefore, the identification of novel actionable biomarkers of liver aging that are both specific and sensitive is the premise for evaluating liver aging and the efficacy of anti-aging interventions, and an outstanding and urgent need in the liver aging field.

The liver, a key hub for numerous physiological processes, continuously maintains metabolic homeostasis for the whole body. Its core functions include energy metabolism, xenobiotic and endobiotic clearance, and molecular biosynthesis. Upon aging, the liver undergoes a series of structural remodeling and functional changes, including decreased liver blood flow, lower regenerative capacity, weaken metabolic function, enhanced numbers of polyploid hepatocytes, and increased immune inflammation and fibrosis [4]. In addition, its capacity to overcome infection and repair injury decreases with aging, which not only increases liver disease risk such as nonalcoholic fatty liver disease (NAFLD), cirrhosis, and liver cancer [5–8] but is also an independent risk factor for primary allograft dysfunction [9, 10].

As a vital endocrine organ in the body, the liver synthesizes and secretes a variety of factors. These factors engage in crosstalk with multiple tissues to systemically affect the physiological state and function of the body. A number of studies have shown that liver-derived factors (hepatokines) play an important role in regulating the energy homeostasis and physiological functions of bone, nervous system, heart, and adipose tissue [11–15]. Therefore, liver aging may be an important factor causing diseases in other organs. In recent years, the magnitude of population aging is increasingly recognized as a serious global problem [16]. According to 2019 population data, the life expectancy of Chinese residents reached 77.6 years, whereas the healthy life expectancy was only 68.4 years [17]. Thus, how to prevent aging-associated diseases, and prolong healthy life expectancy is a monumental challenge in the aging research and the clinical medicine field. Due to liver aging involved in not just liver illnesses but also diseases of other organs, identifying measurable, sensitive, reliable, and specific liver aging biomarkers is needed to assess the degree of liver aging and the efficacy of aging interventions. Such efforts stand to ultimately prevent and treat aging-related diseases, thereby improving the overall healthy life expectancy of the elderly population, and informing formulation of “healthy aging” programs.

On 3 September 2023, the Aging Biomarker Consortium (ABC) [18–24] convened a seminar of experts in the field of liver aging in Beijing, China. Through extensive literature reviews, examination of peer-reviewed research from both domestic and international scientists, evaluation of evidence-based medicine, and incorporation of the unique perspectives of Chinese experts, an expert consensus on biomarkers of liver aging was assembled. The expert consensus aimed to answer pertinent clinical questions such as “What is the biological age of an individual’s liver?,” “What is the rate of liver aging for the individual?,” and “What is the probability that the individual will develop age-related liver diseases?”

## Methods

Literature searches were carried out for studies published before September 2023, and utilizing well-known databases such as MEDLINE, PubMed, and Cochrane Library. By participating in online collaboration, and based on available publications and the collective research conducted by ABC members, the group identified a list of key questions related to liver aging biomarkers. Subsequently, the identified liver aging biomarkers were thoroughly debated to ultimately result in an expert consensus at a validation meeting on liver aging biomarkers held in Beijing on 3 September 2023. All recommendations have undergone a comprehensive review and discussion among ABC members, allowing for multi-dimensional views and considerations elaborated in this consensus document.

This consensus adheres to globally established criteria for describing the level of evidence and strength of recommendations, as shown in Table 1 [25]. Among them, evidence Class I comprises evidence and/or general consensus supporting a particular treatment or procedure that is beneficial, useful, and effective; evidence Class IIa refers to the weight of evidence/opinion in favor of usefulness/efficacy; evidence Class IIb refers to that the usefulness/efficacy is less well established by evidence/opinion; and evidence Class III refers to that the evidence/general agreement suggesting the given treatment/procedure is not useful/effective and sometimes maybe harmful [25].

**Table 1.**
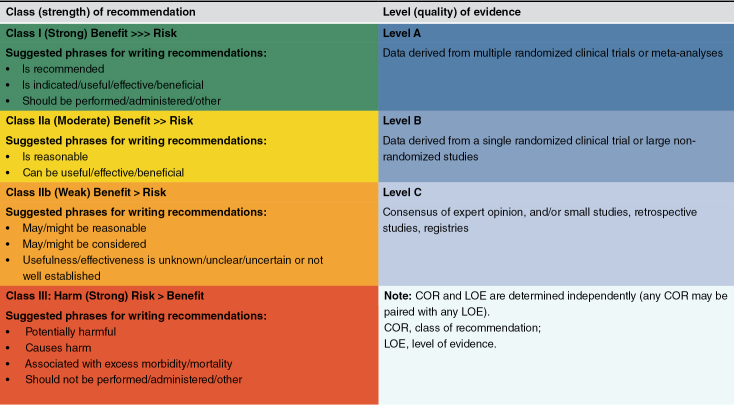
Class of recommendation and level of evidence

## Classification and clinical application of liver aging biomarkers

Liver aging encompasses multi-dimensional and multi-level changes occurring in molecules, cells, organs, organisms, and populations. Liver aging biomarkers denote indicators capable of accurately predicting the “actual age of the liver,” its structural integrity, and functional capacity. These biomarkers serve the purpose of gauging the extent of liver aging and evaluating the effectiveness of interventions aimed at mitigating aging. As an organ with a high metabolic rate in the body, the liver has unique aging characteristics. Therefore, discovering and screening biomarkers related to liver aging is likely to be difficult, requiring verification in large clinical cohorts and careful interpretation of the results. An important issue is how to distinguish physiological liver aging from liver aging induced by pathological states.

Given the considerations of clinical feasibility and convenience, this consensus delineates the screening of liver aging markers across three dimensions: functional, imaging, and humoral. These findings serve as a valuable reference for clinical management and future investigations.

## Functional markers

The liver executes the metabolism, storage, and redistribution of nutrients such as lipids, carbohydrates, and vitamins. The liver is also the main organ for detoxification, and possesses a strong regenerative ability that enables recovery from damage due to toxicity and infection [26]. However, liver aging causes impairments in its regenerative and metabolic capacities, as outlined in more detail below.

### Reduced regenerative capacity

The healthy mammalian liver is endowed with a remarkable regenerative capacity. After partial hepatectomy or chemical injury, hepatocytes regenerate through both cellular hypertrophy and cell division, restoring normal liver function within a short time period [27]. However, the regenerative capacity of the liver declines with age [28, 29], as demonstrated by a clinical study in which the liver regeneration rate showed a significant negative correlation with age in patients with hepatocellular carcinoma one week after hepatectomy surgery [30]. Consistently, an increase in p16 levels and a decrease in expression levels of hepatocyte growth factor and its receptor mesenchymal-epithelial transition factor (c-MET) were reported in the livers of elderly patients with liver tumors aged over 65 years [31]. Although the majority of clinical studies did not detect any significant differences in regenerated liver volume between elderly and young patients after hepatectomy [32–34], these assessments were mostly conducted more than 1 month after surgery, a time point when the liver regenerative process had already been completed. In addition, in the interest of preserving liver function, surgical liver resections are typically controlled to remove a relatively small proportion. Therefore, our insight into regeneration rate in human aging livers after more substantial injury remains limited. Nonetheless, in rodents, a reduced liver regeneration rate associated with aging was reported in the 70% partial hepatectomy model [35–37], and with significantly delayed and reduced expression of cell proliferation-relevant factors [38, 39].

The clinical outcomes of liver transplantation are also partially determined by liver regenerative capacity. In a study that analyzed data from the United States Scientific Registry of Transplant Recipients and the Eurotransplant Registry, high donor age was found to be one of the main factors contributing to high failure rate in liver transplantation [40, 41]. Another study that analyzed 299 cases of living donor liver transplantation surgery found that grafts from older donors generated a smaller volume of regenerated liver than younger donors, at 1 month and 3 months after transplantation [42].

The decline in hepatic regenerative capacity in aging is proposed to be closely related to the increase in hepatocyte polyploidy in the elderly. Most hepatocytes in young adults have low levels of polyploidy, accounting for 6%–15% of total hepatocytes, but hepatocyte polyploidy then accumulates slowly but gradually until after the age of 50 where it reaches 27%–42% [43–46]. Similarly, hepatic polyploidy increased more than threefold in 24-month-old mice when compared to 3-week-old mice, reaching 30%–34% [47, 48]. Hepatocyte polyploidy is also associated with lower proliferative capacity and increased expression of aging markers such as p16, p21, and p53 [43]. In addition, studies also report that reduction in the hepatic regenerative rate in aging livers may be related to factors such as increased oxidative stress, steatosis, apoptosis, and reduced sensitivity to growth factor stimulation in hepatocytes [49–53]. Taken together, the above results suggest that the liver regeneration rate reflects the extent of liver aging; however, it is not recommended as a clinical method to monitor liver aging, given limited sensitivity, and invasive detection approach.

### Impaired drug metabolism

Hepatic clearance of drugs depends on the delivery rate of substrates to drug-metabolizing enzymes within hepatocytes, as well as the intrinsic metabolic capacity of these enzymes [54]. The drug delivery rate, in turn, depends on many key parameters, including liver blood flow, the binding ability between drug compounds and plasma proteins, and the distribution or transfer of drugs from the hepatic blood supply into the space of Disse via the liver sinusoidal endothelial cells.

For drugs with a high hepatic clearance rate (hepatic extraction ratio > 0.7), the rate is approximately equal to that of the hepatic blood flow [54]. However, a number of such drugs have a reduced liver clearance rate in the elderly [55, 56] possibly due to the fact that liver volume and blood flow decrease significantly with age [57, 58].

For drugs with low hepatic clearance rate (hepatic extraction ratio < 0.3), the drug clearance rate is approximately equal to the unbound fraction of the drug in plasma multiplied by the intrinsic hepatic clearance rate [54]. The intrinsic hepatic clearance rate depends mainly on the content and activity of intracellular drug-metabolizing enzymes. Existing research on drug-metabolizing enzymes and aging has mainly focused on the impact of aging on the hepatic clearance rate mediated by the phase I drug metabolic enzymes, primarily cytochrome P450 enzymes [59, 60] as phase II drug metabolism appears to have limited effects on aging [61]. In a study with liver biopsy samples from 226 subjects, the level of cytochrome P450 was found to decrease at a rate of approximately 0.07 nmol/g of liver weight per year after the age of 40 [62]. However, a few studies reported no significant changes in the activity and level of drug-metabolizing enzymes in the aged liver, and that only some cytochrome enzymes were associated with age-related changes in activity [59, 63]. In addition, drugs with low clearance rates showed variable clearing rates in the aged liver [56].

Notably, the aged liver undergoes pseudocapillarization, a phenomenon characterized by thickening of the sinusoidal endothelium with loss of fenestrations, reduced endocytic capacity, and deposition of basal lamina and collagen [64, 65]. Pseudocapillarization results in reduced substrate transfer between blood and hepatocytes, especially in the lobule central vein region [66, 67]. These changes may also reduce liver uptake and clearance of therapeutic drugs associated with macromolecular proteins and liposomes [65, 68, 69]. Taken together, the above results indicate that drug metabolism is significantly impaired in the aged livers, and can serve as a biomarker of liver aging.

### Nutritional and metabolic disorders

#### Lipid metabolism

Liver plays a critical role in maintaining nutritional and metabolic homeostasis. With increasing age, physiological levels of hepatic lipids accumulate, resulting in a higher prevalence of fatty liver disease in the elderly. Multiple population-based cohort studies indicate that the prevalence of fatty liver disease among the elderly population ranges from 35% to 51.4% [70–74]. Contrary to expectations, the prevalence of fatty liver disease diminishes with advancing age within the elderly population. This trend could be attributed to the progression of hepatic steatosis to fibrosis [74]. The overall abnormal lipid metabolism (LM) in the liver to some extent reflects the status of liver aging and thus can be considered as a potential functional biomarker of liver aging.

The lipids chronically accumulating in the liver are mainly triglycerides (TG). Hepatic TG metabolism is related to blood lipid levels, hepatic fatty acid transport, *de novo* fatty acid synthesis, and hepatic lipid output [75]. Blood TG levels are known to increase with age [76], but it is still unclear whether the liver’s ability to take up fatty acids is affected by age. The expression level of fatty acid-binding protein FABP1 in rat livers decreases with age, while the levels of CD36 and SLC27A2, which are responsible for the transport of long-chain fatty acids, increase with age in mouse livers [77–79]. A study including 102 young subjects (22–25 years old) and 170 elderly subjects (62–65 years old) found that FABP4, a fatty acid-binding protein mainly secreted by adipocytes, was significantly upregulated in the plasma of elderly subjects [80]. Consistent with these clinical observations, knocking out *Fabp4* in aged mice significantly attenuated metabolic dysfunction associated with liver aging. Changes in the *de novo* fatty acid synthesis have also been detected in aged livers. Animal experiments have found that sterol regulatory element-binding protein 1 (SREBP1) and carbohydrate-responsive element-binding protein (ChREBP) are likely involved in promoting *de novo* fatty acid synthesis in aged livers [81–83]. The fatty acid oxidation is also an important impact on hepatic TG metabolism. Inducing senescence in mouse hepatocytes led to hepatic lipid accumulation [84], which may be related to mitochondrial dysfunction and reduced fatty acid oxidation capacity caused by hepatocyte senescence [79, 84]. In conclusion, TG metabolism undergoes significant changes in aged livers, but because most of our knowledge stems from animal studies, our understanding of TG metabolism in human remains limited.

Changes in cholesterol metabolism are the most significant and well-studied lipid alterations known to be affected by age. Cholesterol homeostasis is achieved through a balance among cholesterol ingestion, absorption, synthesis, and excretion. Physiological cholesterol needs are met through both dietary intake and endogenous synthesis [85]; however, endogenous *de novo* cholesterol synthesis is the main pathway for the body to obtain cholesterol [86]. The liver is the main organ responsible for synthesizing and clearing excess cholesterol in the body. In animal models, the rate of both cholesterol absorption and hepatic cholesterol level increases with age [87–90]. Consistently, the expression level of the cholesterol synthesis gene *SREBP2* in the livers of aged cynomolgus monkeys are significantly increased, and conversely, SREBP2 overexpression in human primary hepatocytes accelerates cellular senescence [91]. Consistently, in a proteomics study, expression levels of the cholesterol synthase DHCR24 were higher in the livers of the elderly (over 66) relative to young adults (under 47) [92]. In the blood plasma compartment, cholesterol is either in its free form or cholesteryl esters, and constituents of circulating lipoproteins. Part of the cholesterol synthesized in the liver is secreted into the plasma as a component of either high-density lipoprotein (HDL) or very-low-density lipoprotein (VLDL). Hydrolysis of VLDL by lipoprotein lipase and hepatic lipase generates low-density lipoprotein (LDL), the main carrier for transporting endogenous cholesterol. Blood low-density lipoprotein cholesterol (LDL-C) levels increase with age. In the Framingham Study, the average levels of LDL-C were found to steadily increase from 97.08 mg/dL to 132.25 mg/dL in young versus old males, and from 100.44 mg/dL to 156.91 mg/dL in young versus old females, respectively [93]. Under normal circumstances, 65%–70% of LDL in the blood is cleared through the liver’s LDL receptor (LDLR) [94, 95]. However, LDLR levels in the liver decrease with age, thereby reducing the clearance rate of LDL-C [96]. In addition, proprotein convertase subtilisin kexin-9 (PSCK9) interacts with LDLR and promotes its degradation, thereby inhibiting LDL clearance [97]. A study including 2719 Chinese subjects found that the serum level of proprotein convertase subtilisin/kexin type 9 (PCSK9) increased with age [98], presumably one of the factors explaining changes in blood LDL-C and liver LDLR levels associated with aging.

Cholesterol is mainly eliminated from the body through conversion to bile acids in the liver [99, 100]. A study of liver biopsy specimens from 23 subjects showed that the rate-limiting enzyme for bile acid synthesis, CYP7A1, was significantly reduced in elderly subjects and that the serum levels of the bile acid metabolic intermediate 7*α*-hydroxy-4-cholesten-3-one (C4) were also significantly decreased [100]. For every 10 years, it is estimated that the amount of cholesterol converted to bile acids by the body decreases by about 60 mg per day [101]. Given the observed significant changes in the synthesis, secretion, and clearance of cholesterol in aged livers, we recommend the liver’s cholesterol metabolic capacity as a functional biomarker of liver aging.

#### Glucose metabolism

Liver is the main organ responsible for glucose metabolism. In the non-fed state, the liver provides 90%–95% of circulating blood glucose [102]. With increasing age, the body’s ability to regulate glucose metabolism gradually decreases, as evidenced by the Baltimore Longitudinal Study of Aging which found decreased glucose tolerance with increasing age in 2777 healthy subjects [102, 103]. The European Group for the Study of Insulin Resistance used the hyperinsulinemic-euglycemic clamp to measure the endogenous glucose output from the liver in 344 non-diabetic subjects [104]. The results showed that with increasing age, the endogenous glucose output of the liver decreased at a rate of 1.1 ± 0.7 μmol/min per year. However, there was no statistically significant correlation between age and endogenous glucose output. After calibrating the above research data using body mass index, the influence of age on glucose metabolism was greatly reduced. Most studies generally conclude that factors such as weight, exercise, and diet have a more significant impact on glucose metabolism than age [105–107]. Therefore, we do not recommend the level of glucose metabolism in the liver as a functional biomarker of liver aging.

### Imbalanced liver-related coagulation function

Aging is associated with increased levels of coagulation factors and decreased levels of anticoagulant factors [108]. Most coagulation factors are synthesized in the liver. In healthy individuals, plasma concentrations of the coagulation factors I (fibrinogen), V, VII, and IX increase with age [109]. In addition, the plasma fibrinogen concentration increases by about 10 mg/dL every 10 years [110–112]. In addition, the relationship between coagulation factor VII and aging differs between genders such that the level of coagulation factor VII in the blood of elderly women is significantly higher than that in elderly men [113, 114].

The liver is also responsible for the synthesis of anticoagulant factors such as antithrombin III, protein C, and protein S [115]. An analysis of the Third Glasgow MONICA Survey showed that the median antithrombin III level in the blood of elderly men decreased, while the antithrombin III level in women increased after menopause and then gradually decreased with age [116]. For heparin cofactor II (HC II), an anticoagulant enzyme mainly synthesized by hepatocytes, plasma HC II activity decreased with age, and was negatively correlated with the severity of carotid atherosclerosis in a study including 306 subjects aged 40–91 years [117]. Generally, with increasing age, the coagulation factors secreted by the liver increase gradually, whereas the anticoagulant proteins decreased gradually, placing the aging body in a hypercoagulable state. Therefore, we recommend levels of liver-related coagulation factors and anticoagulant enzymes as functional biomarkers of liver aging that can be evaluated by directly detecting coagulation factors and anticoagulant enzymes such as fibrinogen, antithrombin III, etc. in the blood.

### Other functional biomarkers

#### Indocyanine green

Indocyanine green (ICG) is an inert, nontoxic, low-cost water-soluble fluorescent dye. After intravenous administration, ICG is taken up by hepatocytes and secreted into the bile duct and excreted with bile. Therefore, ICG clearance mainly depends on the liver blood flow, the metabolic activity of hepatocytes, and the excretory capacity of bile [118]. The ICG clearance test is commonly used in the clinic to assess liver functional reserve, and dynamic changes in liver function before and after surgery. A large number of population studies have shown that significant changes occur in the blood flow, metabolic level, and bile acid secretion in the liver during aging. Therefore, the ICG clearance test may also offer an important approach to evaluate liver aging. However, the direct evidence supporting the correlation between liver aging and the ICG clearance ability of hepatocytes is still unclear. It is thus recommended to utilizing the capacity of ICG clearance as a promising functional biomarker for assessing liver aging, with strong encouragement to explore the feasibility of incorporating this assessment in future research focusing on aging population cohorts.

#### Mitochondrial dysfunction-related factors

Mitochondria, cellular energy factories, are crucial for maintaining liver function. A large number of studies in humans and rodents have shown that aging leads to mitochondrial dysfunction in the liver. In a study of 107 normal human liver samples, 87% of liver samples from donors over 50 years old showed defects in the mitochondrial respiratory chain, mainly manifested as loss of complex IV expression [119]. Reductions in mitochondrial respiratory chain-related protein activity and expression have been detected in aged rat and mouse liver [120–122]. In old rats, decreases in mitochondrial membrane potential and increases in proton leakage have also been observed in liver tissue [123, 124]. Mitochondria are the main source of endogenous reactive oxygen species (ROS). Compared with young animals, aged rats have significantly increased ROS levels and decreased levels of the critical energy molecule, adenosine triphosphate (ATP), in hepatocytes [123, 125]. Consistently, the aged rat livers are characterized by increased mitochondrial-derived H_2_O_2_ and decreased antioxidant capacity [126]. In conclusion, although the aging liver shows obvious mitochondrial dysfunction, relevant population research data and reliable clinical detection methods remain deficient. Therefore, mitochondrial dysfunction-related factors can be considered as potential functional biomarkers of liver aging that need to be validated.

#### MicroRNA

Aging affects the expression of microRNAs. In an analysis of livers from 4 to 33 months-old mice, microRNAs were reported to be significantly upregulated in aged mice and especially those targeting genes related to liver detoxification and regeneration [127]. In liver biopsy samples from 12 subjects aged 13–87 years, the expression levels of *miR-31-5p*, *miR-141-3p*, *miR-200c-3p*, and *miR-886-5p* were significantly increased in subjects over 70 years old [128]. The *miR-31-5p* and *miR-200c-3p* have the potential to target and be involved in the downregulation of the glutamate transporter *GLT1*, thereby contributing to abnormal glutamine metabolism in aged livers [127, 129, 130]. The expression of other microRNAs is also affected in aged livers. However, their further application requires the population research data and reliable clinical detection approaches for validation Thus, at the present time, evaluating the levels of *miR-31-5p*, *miR-141-3p*, *miR-200c-3p*, and *miR-886-5p* as potential functional and clinical biomarkers of liver aging could be considered.

#### Intestinal microbiota

The liver and intestine are joined bidirectionally via the portal vein and bile duct. The intestinal microbiota provides an initial metabolism of dietary nutrients, which directly affect the absorption of nutrients by liver. Intestinal microbiota is easily affected by factors such as environment, genetic background, and disease conditions. Intestinal microbiota is also known to undergo significant changes with age [131], and intestinal permeability increases with age [132, 133]. These changes may affect the absorption of nutrients by liver, leading to liver inflammation, and accelerating liver aging [134]. Although notable significant differences in the intestinal microbiota between young and old individuals have been identified, our understanding of how these impact on liver aging remains poor. Therefore, whether intestinal microbiota can serve as functional biomarkers of liver aging is of uncertainty and requires further exploration.

#### Recommendations:

(1) The level of cholesterol metabolism in the liver can reflect the degree of liver aging and can be evaluated by assessing the levels of C4, LDL-C, and PCSK9 in the blood (Level C, Class I).
(2) Significant changes occur in some coagulation factors and anticoagulant enzymes synthesized by the aged liver. These can be evaluated by directly detecting the levels of coagulation factors and anticoagulant enzymes such as fibrinogen, coagulation factor VII, antithrombin III , HC II, etc. in the blood (Level C, Class I).
(3) Drug metabolism is impaired in the aged liver. The following drugs with high hepatic clearance rates have reduced hepatic clearance rates in elderly people: amitriptyline, diltiazem, imipramine, labetalol, levodopa, nortriptyline, propofol, propranolol, and verapamil [56]. If patients are taking relevant medications, their hepatic clearance rate levels after taking the medication can be considered for evaluation (Level C, Class IIb).

#### Potential biomarkers:

(1) The ICG clearance test presents a potential approach for assessing liver aging, given its clinical evaluation of liver functional reserve. Thus, the ICG clearance ability is recommended as a potential functional biomarker of liver aging, prompting the need for further research to explore the correlation between liver aging and the ICG clearance ability of hepatocytes in the future.
(2) Aging leads to mitochondrial dysfunction in the liver, but there is a lack of relevant population research data and reliable clinical detection methods. Therefore, factors related to mitochondrial dysfunction can be considered as potential functional biomarkers of liver aging, pending validated.
(3) The expression of several microRNAs is affected in the aged livers, but relevant population research data and reliable clinical detection approaches to validate these microRNAs are currently lacking. Thus, evaluating the levels of miR-31-5p, miR-141-3p, miR-200c-3p*,* and miR-886-5p can be considered as potential functional biomarkers of liver aging.
(4) Intestinal microbiota undergoes significant changes with age, and these changes may affect the absorption of nutrients by liver, accelerate liver aging. However, the study of how intestinal microbiota impact on liver functional aging remains poor. Therefore, intestinal microbiota can serve as potential functional biomarkers of liver aging, necessitating further exploration.

## Imaging biomarkers

There is limited radiological research related to liver aging. Existing evidence suggests that structural imaging, functional imaging, and molecular imaging biomarkers have the potential to reflect liver aging from different perspectives.

### Structural imaging biomarkers

The volume of the liver typically decreases gradually during aging. The liver volume of individuals aged 65 and older is estimated to decrease by 25%–35% compared to those under 40, resulting in a characteristic atrophic appearance of the elderly liver [135]. In a study including 65 healthy men aged 24–91, a significant negative correlation between age and liver volume, as measured by grayscale ultrasound, was reported, showing that at the age of 24, the liver volume per kilogram (kg) body weight was approximately 23.6 mm^3^, while at the age of 91, it was approximately 14.0 mm^3^ [136]. More accurate assessments of liver volume can be achieved through computed tomography (CT) and magnetic resonance imaging (MRI), and these imaging technologies can also be combined with artificial intelligence for rapid and automated quantification of both total liver volume and segmental liver volumes. Single-photon emission computed tomography (SPECT) based on imaging agents that target liver cell asialoglycoprotein receptors can be used for functional liver volume measurement. Wakabayashi et al. conducted SPECT examinations using 99mTc-galactosyl-human serum albumin (99mTc-GSA) in 72 liver tumor patients and found that functional liver volume decreased with increasing age [137].

During aging, certain liver diseases become more common, such as liver cysts, NAFLD, alcoholic liver disease, viral hepatitis, liver fibrosis, cirrhosis, and so on. Various structural imaging techniques can visually display space-occupying lesions such as liver cysts and can also easily reveal hepatic morphological changes related to severe liver fibrosis and cirrhosis [138].

### Functional imaging biomarkers

Liver aging is often associated with a decrease in hepatic blood flow, which can be characterized using pulsed-wave Doppler (PW Doppler) ultrasound or MRI-based blood flow quantification. In a study including 40 healthy adults randomly divided into four age groups (<45 years, 45–60 years, 61–75 years, and >75 years), PW Doppler was used to measure total liver blood flow (the sum of portal vein blood flow and hepatic artery blood flow) and the liver clearance of D-sorbitol were used to measure functional blood flow. The results showed that both total liver blood flow and functional blood flow decreased with age, with a more pronounced decrease in the >75 years group (up to 30%) [139]. Liver aging accompanied by reduced liver blood flow typically involves a decrease in portal vein blood flow, including portal vein velocity and flow volume, while portal vein diameter remains largely unchanged [140]. In a study using MRI-based four-dimensional quantification of portal vein imaging, involving 120 healthy adults aged 30 and above, it was found that peak portal vein flow velocity and flow volume occurred in the 43–44 years age group, with a significant decrease after the age of 60 [141].

Due to thickening of hepatic arteriolar walls, narrowing of lumens, and pseudocapillarization of hepatic sinusoidal endothelial cells, liver aging is typically accompanied by alterations in liver microperfusion. Diffusion-weighted imaging is currently a commonly used MRI functional technique that quantifies the Brownian motion of water molecules and microperfusion within tissues. Pasquinelli et al., in a study including 40 healthy adults (26–86 years old), did not find any statistically significant differences across different age groups in various quantitative parameters related to liver diffusion-weighted imaging, including apparent diffusion coefficient, perfusion fraction, diffusion coefficient (*D*), and pseudodiffusion coefficient (D*) [142]. However, in the context of intra-voxel incoherent motion imaging with the liver, and by exploring signal differences between low *b*-values (between *b* = 0 s/mm² and *b* = 2 s/mm² or between *b* = 0 s/mm² and *b* = 10 s/mm²), Huang et al. introduced biomarker diffusion-derived vessel density (DDVD) as a metric that could characterize liver microperfusion volume and reported that DDVD significantly decreased with age in females [143].

The bullous steatosis of hepatocytes significantly increases with liver aging, and is often accompanied by hepatic iron overload. To quantify liver fat and iron levels, a kind of gradient recalled echo sequence, based on water-fat separation imaging technology (Dixon) and multi-echo calibration, has become the preferred imaging technique and is now widely used domestically. This analysis sequence allows for the simultaneous acquisition of whole liver MRI proton density fat fraction (PDFF) images and R2* images within a single breath hold. The PDFF images can directly measure liver fat content, while the R2* value (1/T2*) is positively correlated with liver iron content. In a large-scale population study including 2561 individuals of German Caucasian descent (1336 females, median age 52 years, interquartile age range 42–62 years), and using a 1.5T magnetic resonance scanner, the median PDFF was found to be 3.9% (range 0.6%–41.5%). Among males, liver fat content continued to increase between the ages of 20 and 50, followed by a decrease thereafter. In contrast, among females, liver fat content remained relatively stable before the age of 40, increased continuously between the ages of 40 and 65, and then decreased [144]. The study results also showed that the median R2* value in the liver was 34.4 s^−1^ (range 14.0–311.8 s^−1^). Using an R2* > 41 s^−1^ threshold as the standard for iron overload, 17.4% of participants were found to have liver iron overload, with a significantly higher prevalence in males (27.2%) compared to females (9.0%) [144].

Liver aging may be accompanied by liver fibrosis, the diffuse excessive deposition and abnormal distribution of extracellular matrix components such as collagen, glycoproteins, and proteoglycans. Conventional ultrasound, CT, and MRI technologies fail to detect early stage liver fibrosis and are, therefore, unsuitable for monitoring age-related liver fibrosis. In contrast, transient elastography and magnetic resonance elastography (MRE) are promising noninvasive methods for the diagnosis and assessment of liver fibrosis, applicable to detect liver fibrosis and useful for staging liver fibrosis. For example, the area under the receiver operating characteristic curve for MRE in diagnosing chronic hepatitis liver fibrosis stages ≥ F1, ≥ F2, ≥ F3, and F4 is 0.84, 0.88, 0.93, and 0.92, respectively [145]. However, MRE is currently limited to clinical research. It was reported that three-dimensional imaging of the spin-lattice relaxation time in the rotating frame (T1ρ) with MRI could effectively detect early liver fibrosis, as T1ρ prolongation served as a sensitive marker for collagen fiber deposition and could distinguish between no liver fibrosis and F1 stage fibrosis in experimental mice [146]. However, this technology has not yet been commercially applied.

### Molecular imaging biomarkers

In liver fluorine-18-fluorodeoxyglucose (18F-FDG) positron emission tomography/computed tomography (PET/CT) examinations, the maximum standard uptake value (SUV) and average SUV of the liver increase with age. In a study that included 2526 18F-FDG PET/CT scans, age emerged as a crucial predictor of liver SUVs, showing a notable association with both the maximum SUV (*β* = 0.347, *P* = 0.000) and the average SUV (*β* = 0.354, *P* = 0.000). The liver SUVs exhibited a swift ascent until the age of 20, followed by a gradual upward trend without reaching a plateau [147]. Another study also confirmed that adult liver metabolic activity increased with age and that there was a positive correlation between the two (*r* = 0.4434, *P* = 0.0029). However, the liver metabolic volume (defined as liver volume multiplied by the average SUV) did not significantly change with age [148].

#### Recommendations:

(1) The degree of hepatic steatosis can serve as an imaging biomarker for aging-related liver fat deposition. Assessment can be performed using MRI-PDFF (Level A, Class IIa).
(2) Liver relative volume can serve as an imaging biomarker for assessing liver aging, and the degree of volume reduction can help predict the status of liver aging. Clinically, liver volume can be evaluated through liver CT or MRI, and artificial intelligence technology can be combined for automated and rapid assessment (Level C, Class IIa).
(3) Liver blood flow can serve as an imaging biomarker for assessing liver aging, and a reduction in blood flow can help predict the status of liver aging. Liver blood flow assessment can be performed using PW Doppler or MRI (Level C, Class IIa).

## Humoral biomarkers

Components in bodily fluids such as blood, urine, and bile have become indispensable biological markers for assessing liver aging due to their noninvasive or minimally invasive nature, high sensitivity, and ease of accurate measurement. This consensus framework is aimed at recommending biomarkers related to liver aging, thus, the strategy for searching for biomarkers in body fluids is to screen for liver tissue cell-specific markers that are highly likely to be correlated with the level of liver aging.

### Liver function related markers

#### Albumin

Albumin is the main protein in human plasma synthesized by the liver, which maintains the body’s nutrition and osmotic pressure and can reflect the body’s nutritional status and liver function. However, studies reporting on serum albumin variation levels in aging animals are not consistent [149]. In a small-sample study of a normally aging human population, serum albumin levels were found to slightly decrease with age [76]. Due to a lack of more clinical evidence, whether serum albumin concentration can serve as a specific marker for assessing the levels of liver aging still needs to be verified by follow-up cohort studies, but it can be considered as a candidate marker for liver aging.

#### Lipids

Lipids include TG, phospholipids, and sterols, primarily synthesized by the liver. In a small-sample cohort study, serum total cholesterol (TC), HDL-cholesterol (HDL-C), and TG levels were reported to increase with age [76]. In another cohort study, LDL-C metabolism in the elderly population was reported to decline by 35%, leading to an increase in plasma LDL-C levels with age [150]. These changes in blood lipids have also been validated in some aged animal model studies [151, 152].

Although TC and TG concentrations in the blood increase with age, the TG synthesis and metabolism in the body are not only carried out in the liver but also in the small intestine and adipose tissue, which play a crucial role. The fat distribution and storage in the body and nutritional status and lifestyle directly affect TG levels in the blood. Additionally, apart from the liver, nutritional status and intestinal absorption also have a significant impact on TC levels in the blood. Therefore, future cohort studies are required to establish whether specific lipid changes in the blood can be considered as body fluid biomarkers for assessing liver aging.

Furthermore, clinical research has found serum levels of PCSK9, an enzyme related to liver metabolism, increase with age [98], while the content of C4, an intermediate in bile acid metabolism, significantly decreases in the elderly population [100]. Hence, both PCSK9 and C4 can be considered as body fluid markers for assessing liver aging.

Finally, it is important to note that the level of lipid synthesis and secretion in the liver is closely related to individual genetic background differences and liver metabolism-related diseases. Therefore, when using specific lipids as predictive biomarkers for liver aging, they need to be differentiated from these pathological conditions.

#### Apolipoproteins

Apolipoproteins, a protein component constituting plasma lipoproteins, are divided into five classes: A, B, C, D, and E. They are primarily synthesized by the liver (partially by the small intestine) and can bind to cholesterol or other lipids to form lipoprotein particles, thereby mediating lipid transportation. Studies have found that Apolipoprotein E (APOE) is upregulated in aging animal liver tissues and in various human aging stem cell models. Consistently, overexpression of APOE can accelerate the aging of human stem cells (mesenchymal progenitor cells), while APOE knockout mediated by CRISPR/Cas9 can slow down human stem cell aging. These findings suggest that the accumulation of APOE may be a new driving force of cellular aging [153, 154].

A multi-dimensional study of aging biomarkers in a natural population cohort of Chinese women aged 20–66 established in Quzhou, Zhejiang Province, found a high positive correlation between aging and the upregulation of apolipoprotein APOE, APOC4 in plasma [155]. Since apolipoproteins are mainly synthesized by the liver, the elevation of apolipoproteins may to a certain extent indicate the degree of human liver aging. Therefore, we suggest that apolipoprotein APOE and APOC4 can be considered as body fluid markers for liver aging.

#### Liver enzymes

Liver enzymes, such as alanine aminotransferase (ALT), aspartate aminotransferase (AST), alkaline phosphatase (ALP), and gamma-glutamyl transferase (GGT), detected in liver serological biochemical tests, are important indicators of liver damage. Studies on aging animals have found that serum levels of ALT, AST, and ALP increased with age [156]. However, in a small-sample cohort study (327 cases) of aging individuals, serum transaminase levels (ALT, AST) essentially remained normal, while levels of ALP and GGT increased with age [76]. In another slightly larger cohort study (1673 cases), specifically targeting aging male groups over 70 years old, ALT levels were found to be significantly decreased with age, and the decrease in ALT levels was associated with frailty and decreased survival rate [157].

Due to the inconsistency of results from different clinical cohort studies, it remains to be determined if liver enzymes, especially ALT, ALP, and GGT, can serve as specific markers to assess liver aging. However, they can be considered as candidate biomarkers in body fluids for assessing liver aging. Moreover, serum liver enzyme levels are closely related to various liver diseases. Thus, when used as predictive biomarkers for liver aging, liver enzymes need to be differentiated from liver diseases.

#### C-reactive protein

The C-reactive protein (CRP), structured as a pentamer and produced by liver cells, is an acute-phase protein serving as a nonspecific marker for inflammation and tissue damage. In a large sample study of healthy aging (without significant cardiovascular diseases, myocardial infarction, stroke, type 2 diabetes, and cancer) blood CRP levels were found to increase in an age-dependent manner. Higher CRP levels were also associated with reduced survival rates, and worsened physical performance, and cognitive abilities [158]. In a second study that included 2944 healthy women aged 30–79, CRP levels were reported to increase in an age-dependent manner [159].

Additionally, a longitudinal study over 10 years, following the CRP levels of healthy aging individuals in the UK, found that the risk of adverse aging outcomes increased for elderly individuals who experienced an increase in CRP level over the 10-year period [160]. A large cohort study for the healthy aging population in China also suggested that serum (high sensitivity) CRP levels could be an indicator of healthy aging [161]. Thus, several cohort studies conclude that increased blood CRP levels are closely related to systemic aging. In a recent study in non-human primate aging models, the transcriptional level of CRP in aging liver cells was reported to be increased [91], adding to the body of research indicating that an increase in CRP levels also reflects liver aging. Therefore, we recommend considering CRP a candidate biomarker for liver aging, but note that its assessment needs to be differentiated from infections and injuries in the body.

#### Insulin-like growth factor 1

Insulin-like growth factor 1 (IGF-1) is a growth hormone-regulated endocrine hormone secreted by the liver that mainly regulates cell growth and metabolic synthesis. After birth, IGF-1 levels increase along with an increase of growth hormone secretion, continue to rise steadily during childhood, and increase significantly before puberty. However, multiple large cohort studies show that after age 20, serum IGF-1 levels decline linearly with increasing age, and that the rate of decline in women under 55 years is more pronounced than in men [162–164].

Although serum IGF-1 levels are closely related to age, liver IGF-1 secretion is significantly regulated by growth hormone. Therefore, when considering IGF-1 as a marker of liver aging, the effect of growth hormone secretion levels should be taken into account and IGF-1 tested in combination with other liver aging markers.

#### Transthyretin protein

Transthyretin (prealbumin) is synthesized in the liver and serves to transport retinol-binding protein–vitamin A complex and thyroxine in the bloodstream [165]. In cohort studies, the protein level of transthyretin is reported to be more sensitive than albumin in reflecting acute liver injury [166], and follow-up cohort studies could help verify whether transthyretin can be used as a marker to characterize the level of liver aging. It is worth noting that the change in transthyretin level is also related to the nutritional status of the body and liver damage caused by various reasons, which would be important to be further comprehensively evaluated when using serum prealbumin levels as a biomarker for predicting liver aging.

### Biomarkers unrelated to liver function

#### Osteopontin

Osteopontin (OPN) is a glycosylated extracellular matrix protein expressed by various tissue cells and recently become recognized as an inflammation-related factor. In a small-sample cohort study, serum OPN levels were found to increase with age. Although this trend was not detected in NAFLD groups of different ages, both serum and liver tissue OPN levels in experimental studies of aging animals produced results that were consistent with findings in older people [167]. OPN deficiency increases sensitivity of the liver to aging and liver aging-related diseases, and exacerbates liver fat accumulation and fibrosis, indicating that OPN is protective against aging. Although OPN can be considered a candidate biomarker in body fluids for liver aging assessment, its specificity as a marker for assessing levels of liver aging requires verification in additional cohort studies. Furthermore, increased OPN levels are also associated with biliary diseases and some inflammatory diseases. Therefore, when considered as a predictive biomarker for liver aging, OPN will need to be combined with other biomarkers.

#### Mesencephalic astrocyte-derived neurotrophic factor

Mesencephalic astrocyte-derived neurotrophic factor (MANF) is an evolutionarily conserved protein associated with repair of tissue injury and alleviation of inflammation, and detected in human blood. In a small-sample cohort study, MANF serum levels decreased with age [168], and similar findings were documented in aged fly and aged mouse tissues (including liver tissue). In parabiotic mouse models, MANF derived from young mice was found to be necessary for liver rejuvenation in aged mice. Furthermore, age-related decrease in MANF levels is associated with disruption of tissue metabolic homeostasis, while systemic MANF supplementation can extend the lifespan of flies, relieve symptoms of metabolic disorders and inflammation related to liver aging in mice, and prevent diet-induced liver steatosis, suggesting that MANF is a circulating anti-aging factor. Therefore, MANF can be considered a candidate serum biomarker for assessing liver aging. However, to specifically assess liver aging levels, MANF must be used in combination with other liver aging biomarkers, as various tissue cells in the body, such as neurons, immune cells, liver cells, etc., can secrete MANF into the blood.

#### Chitotriosidase-3-like protein 1

Chitotriosidase-3-like protein 1 (CHI3L1), also known as chitinase, is a secreted glycoprotein and pro-inflammatory factor. Recent studies in China and other countries have found that the expression level of CHI3L1 in the blood can help assess the degree of liver fibrosis, proposing CHI3L as a new marker of liver fibrosis [169]. In a recent Chinese non-human primate aging model, CHI3L1 transcriptional levels specifically increase in aging liver cells, consistent with the earlier discovery of CHI3L1 trends in serum proteomics of different age groups [91]. These results suggest that CHI3L1 can be considered a candidate marker for liver aging upon further verification in subsequent cohort studies. Similarly, when CHI3L1 is employed as a marker of liver aging, it should be distinguished from the markers of liver fibrosis since it plays a role in liver fibrosis.

#### Leukocyte DNA methylation

In eukaryotes, DNA methyltransferases DNMT1, DNMT3a, and DNMT3b conduct most DNA methylation (DNAm) at the 5th carbon position of cytosine, forming CpG dinucleotides. After the age of 60, liver epigenetic aging is mainly manifested by the downregulation of DNA methyltransferase, that is, a general decrease of DNA methylation throughout the entire genome, leading to activation of silent genes and other DNA sequences [170, 171]. DNA methylation changes associated with liver aging mainly manifest at CpG sites in white blood cells, such as cg16867657 in the promoter of the *ELOVL2* gene, which encodes fatty acid elongase 2 and is closely related to human liver aging [172].

Despite insufficient clinical evidence, and the need for subsequent cohort studies to further ascertain whether DNA methylation can serve as a specific marker for liver aging, it is worth considering leukocyte DNA methylation in body fluids as a candidate biological marker for assessing liver aging.

#### Senescence-associated secretory phenotype molecules

The senescence-associated secretory phenotype refers to pro-inflammatory factors secreted by cells upon aging. Aging liver cells and bile duct cells express higher levels of IL-6, IL-8, TNF-α, etc., and pro-inflammatory factors are known to cause chronic inflammatory responses associated with liver aging [4]. However, these inflammatory markers are broad markers for both inflammatory diseases and various liver diseases, making them unsuitable to be used solely as liver aging biomarkers.

#### Recommendations:

(1) Serum albumin levels, especially when lower, exhibit a potential as a biomarker for predicting liver aging (Level B, Class IIa).
(2) TC, HDL-C, LDL-C, TG, PCSK9, and C4 with increased levels may act as predictive biomarkers for liver aging (Level B, Class IIa).
(3) Increased levels of the plasma apolipoproteins APOE and APOC4 suggest a higher likelihood of liver aging and may be utilized as potential biomarkers for this condition (Level B, Class IIa).
(4) Changes in serum levels of ALT, ALP, and GGT, specifically a decrease in ALT and increasing levels in ALP and GGT, could potentially be considered as biomarkers for assessing liver aging (Level B, Class IIa).
(5) Increased levels of blood CRP may serve as a biomarker for predicting liver aging (Level B, Class IIa).
(6) Decreased levels of serum IGF-1 may suggest liver aging. However, the influence of changes in growth hormone, which directly affects IGF-1 production should be taken into account simultaneously (Level B, Class IIa).
(7) Elevated serum OPN levels could potentially be considered as biomarkers for assessing liver aging. However, conclusive evidence of their predictive value requires (Level C, Class IIb).
(8) Serum MANF levels could be considered as potential biomarkers for liver aging, with decreased levels possibly indicating liver aging. To firmly establish the role of serum MANF levels in the assessment of liver aging, it is necessary to conduct subsequent cohort studies. (Level C, Class IIb).
(9) Elevated Serum CHI3L1 levels could be potential biomarkers for liver aging. Conducting cohort studies to evaluate the sensitivity, specificity, and predictive value of CHI3L1 levels concerning liver aging is crucial for validation (Level C, Class IIb).
(10) An increased methylation level of cg16867657 in the promoter region of leukocyte *ELOVL2* could potentially serve as a biomarker for assessing liver aging. Subsequent cohort studies are required for confirmation (Level C, Class IIa).

#### Potential biomarkers:

(1) Transthyretin is reported to be sensitive in reflecting acute liver injury, but its relationship with liver aging has not been elucidated. Thus, follow-up cohort studies should be organized to verify whether transthyretin can be used as a marker to characterize the level of liver aging.

## Building models to assess and predict liver aging and liver aging-related diseases

Liver aging includes a series of changes from molecular to functional levels. Therefore, the accurate, consistent, and comprehensive assessment and prediction of liver aging and its associated diseases requires the integration of data derived from diverse markers operating across multiple scales, dimensions, and modalities. In recent years, significant progress has been made in exploring the biological age of the liver (hereinafter referred to as “Liver Age”). Biological age is inferred from the physiological status of normal human organismal biology and anatomy. As the primary cause of aging-related conditions, biological age has proven to be superior to chronological age in terms of accurately measuring real differences in aging among individuals. However, the field of aging research has not yet reached a consensus on a quantitative method for assessing biological age. The liver aging biomarkers described above represent quantifiable indicators for accurate assessment of liver age; conversely, liver age can also provide a standardized evaluation system for the study of liver aging biomarkers. In addition, the evaluation of liver age is of great significance for studying mechanisms and intervention strategies in liver aging and liver aging-related diseases. Thus, the evaluation of liver age stands to become pivotal for the early identification of pathological liver aging and the accurate evaluation of therapies targeting liver aging-related diseases.

The rise of machine learning has promoted research on a “liver age assessment model.” Liver aging is mainly manifested in reduced liver volume, reduced blood flow, and decreased liver function [4, 46]. At present, the construction of liver age is mostly based on MRI and biological measurement of liver function [137]. Although these “liver age assessment models” based on radiomics and liver function data have great potential and applicability in basic and clinical research, the existing models still lack validation and optimization of long-term follow-up data based on multi-center large sample size.

The difference between the predicted liver age and the chronological age based on the model evaluation is called the “liver age gap (LAG)” [173, 174], in which the LAG reflects whether an individual liver has accelerated or delayed aging. As such, the LAG provides an important reference for predicting the rate of individual liver aging, offering an early alert for potential risks associated with the development of liver aging-related conditions, such as increases in NALFD and cirrhosis with advancing age [5, 6, 175]. However, an accurate, well-developed, and widely accepted assessment model for liver aging is still exploring. At present, a study using deep learning has built a liver age predictor by training convolutional neural networks and found that the liver age difference of patients with cirrhosis can reach more than 4 years old [174], but there is still a lack of long-term follow-up studies of large samples and a specific, multi-dimensional biomarker calculation model in the construction of liver age assessment model and liver aging-related disease prediction model. In addition, establishing an accurate, broad-applicated liver aging-related disease prediction also need combine liver aging biomarkers and disease-specific markers for comprehensive analysis.

Liver aging and functional degradation involve changes at multiple levels from molecules to cells to organs to systems. Applying LAG, therefore, holds great significance for identifying aging-related liver health problems and formulating strategies to prevent liver aging-related diseases. Similarly, liver age assessment model and liver disease prediction model based on multimodal biomarkers applied towards identifying early warning signs of liver aging and liver aging-related diseases and informing future research directions, stand to become more powerful when combined with in-depth application of machine learning and artificial intelligence models in biomedicine.

## Conclusion and future perspectives

### Key recommended biomarkers of liver aging

**Figure 1.**
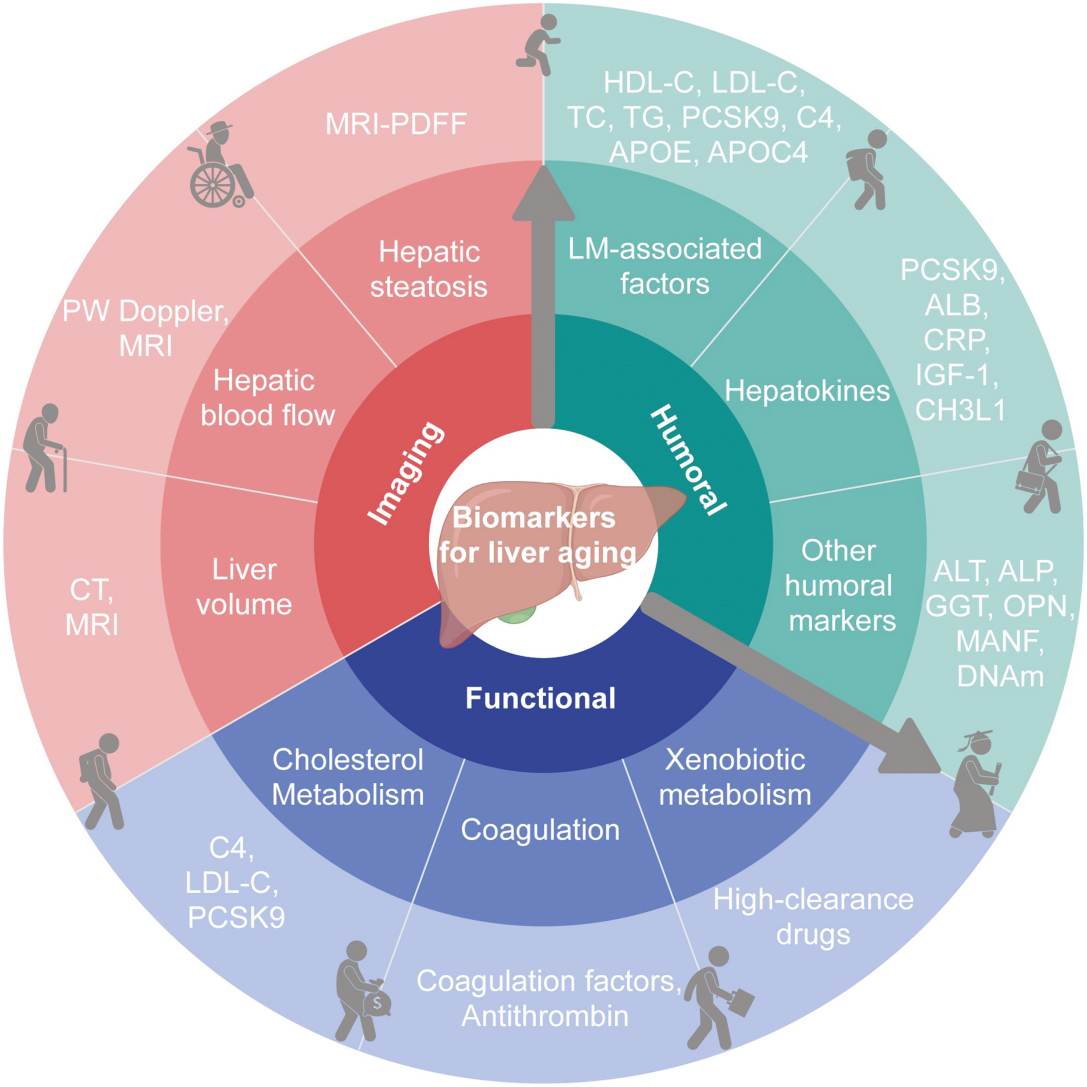
**Framework of biomarkers for liver aging.** The proposed framework for liver aging consists of three dimensions: functional, imaging, and humoral biomarkers. Abbreviations: ALP, alkaline phosphatase; ALT, aminotransferase; APOC4, apolipoprotein C4; APOE, apolipoprotein E; C4, 7α-hydroxy-4-cholesten-3-one; CHI3L1, chitotriosidase-3-like protein 1; CRP, C-reaction protein; CT, computed tomography; DNAm: DNA methylation; GGT, gamma-glutamyl transferase; HC II, heparin cofactor II; HDL-C, high-density lipoprotein cholesterol; IGF-1, insulin-like growth factor 1; LDL-C, low-density lipoprotein cholesterol; LM, lipid metabolism; MANF, mesencephalic astrocyte-derived neurotrophic factor; MRI, magnetic resonance imaging; OPN, osteopontin; PCSK9, proprotein convertase subtilisin/kexin type 9; PDFF, proton density fat fraction; PW Doppler, pulsed-wave Doppler; TC, total cholesterol; TG, triglycerides.

According to the expert discussion, the following 16 liver aging biomarkers are recommended from 3 dimensions: functional, imaging, and humoral factors (Fig. 1, Table 2). In view of their broad clinical applicability, the most strongly recommended liver aging biomarkers include alterations of cholesterol metabolism, liver-related coagulation function, hepatic steatosis, liver blood flow, and hepatokines (Table 2).

**Table 2.**
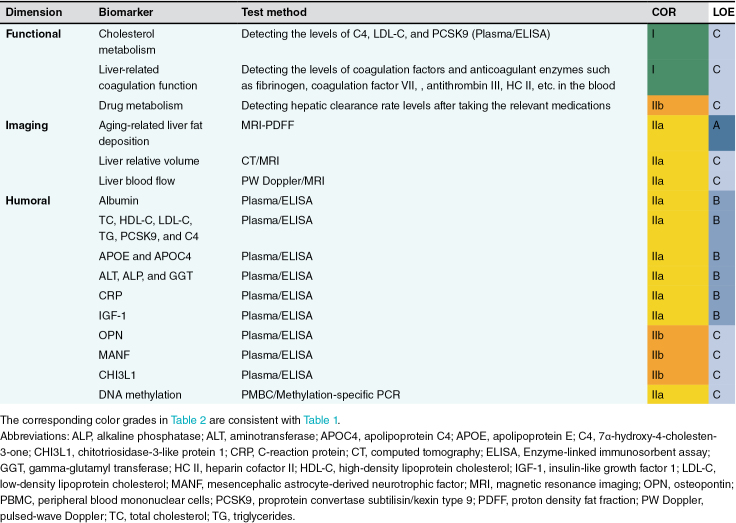
Recommended biomarkers of liver aging

### The working route map of liver aging biomarker research

The expert consensus summarized liver aging biomarkers and potential biomarkers, and these biomarkers need to be validated in a leverage high-quality population-based research in the future. This will provide guidance for the evaluation of liver aging and liver aging-related diseases, speed up the research process of liver aging intervention, and contribute to the healthy aging of the human liver.

The action framework for liver aging biomarker research in China includes the following objectives: (1) To establish a national multi-center aging cohort for the “1000 Individuals Liver Aging Research Program” within China: discover and verify liver aging biomarkers, establish detection techniques and methods, and determine the reference values of liver aging biomarkers in Chinese population, predict the age “inflection point” of liver aging, and to clarify the individualized intervention time window of liver aging and liver aging-related diseases. (2) To use artificial intelligence to establish a liver aging assessment model and a liver aging-related diseases prediction model. (3) To promote the deep collaboration among industry, academia, research, and government, and encourage the application and transformation of scientific findings. The establishment of liver aging biomarker system will help to promote the clinical and basic research of liver aging-related diseases, and ultimately improve the health level of the elderly population in China.
